# Diagnostic value of ratio of blood inflammation to coagulation markers in periprosthetic joint infection

**DOI:** 10.1515/med-2025-1150

**Published:** 2025-02-27

**Authors:** Jincheng Huang, Xu Li, Yajun Chen, Meng Zhang, Zongyan Gao, Zhipeng Dai, Tao Liu, Yi Jin

**Affiliations:** Department of Orthopedics, Henan Provincial People’s Hospital, People’s Hospital of Zhengzhou University, People’s Hospital of Henan University, Zhengzhou, Henan, 450003, P. R. China; Department of SICU, Zhengzhou First People’s Hospital, Zhengzhou, Henan, 450000, P. R. China; Department of Orthopedics, Henan Provincial People’s Hospital, People’s Hospital of Zhengzhou University, People’s Hospital of Henan University, No. 7, Road Weiwu, Zhengzhou, Henan, 450003, P. R. China

**Keywords:** periprosthetic joint infection, D-dimer, fibrinogen, platelet count and mean platelet volume ratio (PC/MPV), fibrinogen, ratio

## Abstract

**Introduction:**

Assess the feasibility of utilizing the ratio of blood inflammation to coagulation markers as a potential periprosthetic joint infection (PJI) diagnostic tool.

**Materials and methods:**

A retrospective analysis was conducted, involving 133 PJI and 93 aseptic loosening patients. Levels of C-reactive protein (CRP), erythrocyte sedimentation rate (ESR), platelet count, mean platelet volume, fibrinogen, D-dimer, and ratios of CRP to fibrinogen, ESR to fibrinogen, platelet count and mean platelet volume ratio (PC/MPV), and D-dimer were compared. Receiver operating characteristic curves and Youden’s index were employed to assess the diagnostic efficacy of these biomarkers.

**Results:**

PJI patients had significantly higher levels of CRP, ESR, PC/MPV ratio, fibrinogen, D-dimer, CRP/(PC/MPV) ratio (CPR), CRP/D-dimer, CRP/fibrinogen (CFR), ESR/(PC/MPV) ratio, ESR/D-dimer, and ESR/fibrinogen. Area under the curve (AUC) values for fibrinogen, CRP, and ESR in diagnosing PJI were comparable. AUC values for CPR and CFR were akin to those of ESR. AUC values for combined CRP and CPR, combined CRP and fibrinogen, combined CRP and CFR, and combined ESR and fibrinogen in diagnosing PJI were akin to that of combined CRP and ESR.

**Conclusions:**

Fibrinogen, CPR, CFR, combined CRP and CPR, combined CRP and fibrinogen, combined CRP and CFR, and combined ESR and fibrinogen could be considered as new adjunct markers for diagnosing PJI.

## 1 Introduction

While C-reactive protein (CRP) and erythrocyte sedimentation rate (ESR) are commonly recommended blood inflammation markers for diagnosing periprosthetic joint infection (PJI) according to several PJI diagnosis guidelines [1,2,3,4,5], their performance may be suboptimal in certain situations, such as in cases of low-grade virulence or chronic PJI [6]. Thus, the search for new adjunct markers for PJI diagnosis remains a critical focus in current orthopedic research [7,8,9,10,11]. Building on the coagulation and inflammation theory [12], various authors have investigated the roles of commonly used coagulation markers, such as D-dimer [7], fibrin degradation product (FDP) [13], platelet count and mean platelet volume ratio (PC/MPV) [14], and fibrinogen [8,15,16] in PJI diagnosis over the past few years. However, the significance of D-dimer and PC/MPV ratio in PJI diagnosis remains a topic of debate [14,17,18,19]. While individual inflammation or coagulation markers may contribute to PJI diagnosis, the utility of the ratio of inflammation to coagulation markers for this purpose has been underexplored [20]. Understanding the precise value of the ratio of inflammation to coagulation markers in PJI diagnosis may offer additional adjunct markers for PJI diagnosis. This study aims to: (1) evaluate the utility of traditional inflammation or coagulation markers in PJI diagnosis when used individually; (2) assess the significance of the ratio of inflammation to coagulation markers in PJI diagnosis; and (3) appraise the value of combined inflammation and the ratio of inflammation to coagulation markers in PJI diagnosis.

## 2 Materials and methods

### 2.1 Study design and settings

The retrospective study design consisted of patients diagnosed with PJI and aseptic loosening at our department from January 2017 to December 2022. This retrospective study received approval from the Ethics Board of Henan Provincial People’s Hospital and adhered to the principles of the Declaration of Helsinki. Given the retrospective design of the study, the requirement for informed consent was waived by the Ethics Board of Henan Provincial People’s Hospital.

### 2.2 Study protocol

After the ethics committee approval was received, the data on the hospital’s data network were retrospectively examined for patients diagnosed with PJI and aseptic loosening. Clinical data, including sex, age, duration of follow-up, type of affected joints, preoperative CRP, ESR, D-dimer, PC/MPV ratio, fibrinogen, CRP/D-dimer (CDR), CRP/(PC/MPV) ratio (CPR), CFR (CRP/fibrinogen), ESR/D-dimer (EDR), ESR/(PC/MPV) ratio (EPR), and ESR/fibrinogen (EFR) levels, were evaluated.

The exclusion criteria were defined as follows: (1) presence of visible ecchymosis; (2) history of recent dislocation or trauma (within 2 weeks); (3) presence of hematoma or any type of skin ulcer; (4) history of hypercoagulation disorder; (5) presence of a prosthetic heart valve; (6) diagnosis of a systemic inflammatory disease; and (7) history of tumor. Detailed information on these exclusion criteria can be found in our previously published paper [17].

PJI was defined in accordance with the criteria set forth by the Musculoskeletal Infection Society (MSIS) [21]. Aseptic loosening was defined based on the following criteria: (1) presence of thigh or hip region pain and knee pain; (2) radiological evidence of loosening (such as disintegration of prosthesis components with the bone, displacement of prosthesis components, or circumferential radiolucent line); and (3) absence of criteria indicative of PJI.

### 2.3 Data analysis

Quantitative data were presented as mean ± standard deviation, and comparisons between multiple groups were conducted using a single-factor analysis of variance. The Student–Newman–Keuls test was employed for normally distributed data, while Tamhane’s *T*2 test was utilized for non-normally distributed data when comparing two means. The chi-square test (*χ* ^2^) was used for comparing categorical data among groups. Statistical significance was set at *P* < 0.05. Statistical analyses were performed using IBM SPSS Statistics version 19 (IBM SPSS Software).

The performance of CRP, ESR, D-dimer, PC/MPV ratio, fibrinogen, CDR, CPR, CFR, EDR, EPR, EFR, as well as combinations of CRP or ESR with CDR, CPR, CFR, EDR, EPR, or EFR, was evaluated through receiver operating characteristic (ROC) analyses using MedCalc 19.0.4 (MedCalc Software, Ostend, Belgium). Parameters such as sensitivity, specificity, and the area under the curve (AUC) were assessed, with an AUC of >0.7 considered acceptable. Optimal thresholds were determined using Youden’s index. DeLong’s test was used to compare the AUC of different markers in PJI diagnosis.


**Informed consent**: Given the retrospective nature of the study and the use of de-identified patient data, the requirement for informed consent was waived by the Ethics Board of Henan Provincial People s Hospital. The study was conducted in accordance with the ethical standards of the Declaration of Helsinki and its later amendments.
**Ethics approval:** This retrospective study was approved by the Ethics Board of Henan Provincial People’s Hospital.

## 3 Results

### 3.1 Demographic characteristics of the enrolled patients

**Figure 1:**
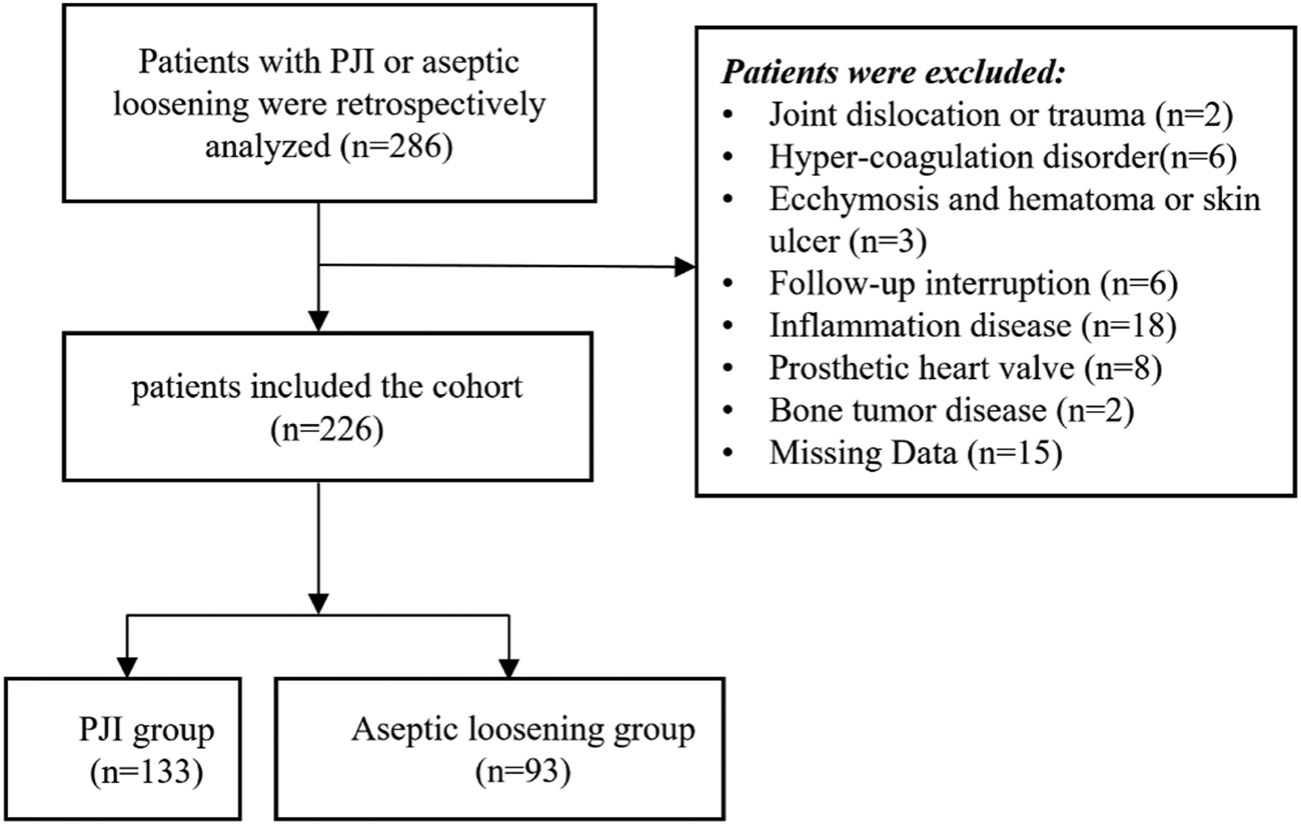
Flow diagram of included patients.

A total of 286 patients presenting with PJI or aseptic loosening at our department between January 2017 and December 2022 were included in this retrospective study. Fifteen cases were excluded due to incomplete demographic, clinical information, and laboratory results. Two cases were excluded due to a recent history of dislocation or trauma (within 2 weeks), while 6 cases were excluded due to a history of hypercoagulation disorder. Three cases were excluded due to visible ecchymosis, hematoma, or skin ulcer, and 18 cases were excluded due to a history of systemic inflammatory disease. Additionally, eight cases were excluded due to the presence of a prosthetic heart valve, six cases due to interrupted follow-up, and two cases due to primary joint arthroplasty for bone tumor disease. Ultimately, 226 patients met the inclusion and exclusion criteria for this study (Figure 1). All patients were followed up for at least 2 years. The patients’ demographic details were recorded in an electronic database (Table 1). There was no significant difference when compared with the sex, age, and duration of follow-up between patients from the two groups, while there was a significant difference when compared with the type of affected joints between patients from the two groups.

**Table 1:** Demographic characteristics of the enrolled patients

Parameters	PJI (*n* = 133)	Aseptic loosening (*n* = 93)	*p*-value
**Sex,** ***n*** **(%)**			0.181*
Male	59 (44.36)	33 (35.48)	
Female	74 (55.64)	60 (64.52)	
Affected joints, ***n*** **(%)**			<0.001*
Knee	68 (51.13)	19 (20.43)	
Hip	65 (48.87)	74 (79.57)	
**Mean age, years (SD)**	64.74 (12.09)	65.94 (9.63)	0.427†
**Duration of follow-up, months (SD)**	51.67 (14.33)	49.57 (16.38)	0.310†

*Chi-squared test.

†Independent-samples *t*-test.

### 3.2 Value of fibrinogen, D-dimer, PC/MPV ratio, CPR, CDR, CFR, EPR, EDR, and EFR in PJI diagnosis

**Figure 2:**
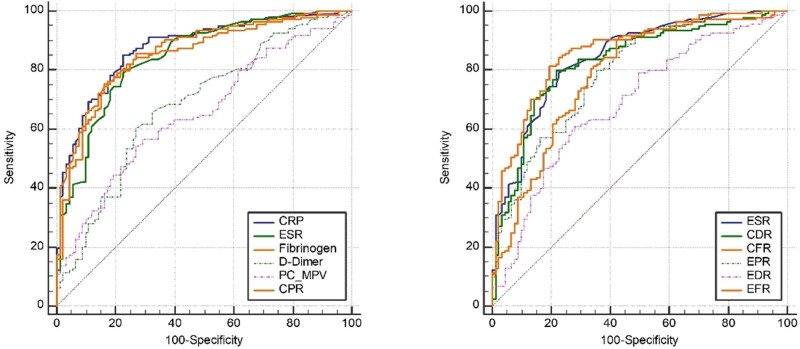
ROC curve of CRP, ESR, fibrinogen, and ratio of inflammation to coagulation markers in PJI diagnosis.

In the initial analysis, levels of CRP, ESR, PC/MPV ratio, fibrinogen, D-dimer, CPR, CDR, CFR, EPR, EDR, and EFR were compared between patients in the two groups. As depicted in Table 2, the PJI group exhibited significantly elevated levels of CRP, ESR, PC/MPV ratio, fibrinogen, D-dimer, CPR, CDR, CFR, EPR, EDR, and EFR compared to the aseptic loosening group. Tables 3 and 4 and Figure 2 illustrate that fibrinogen demonstrated diagnostic performance comparable to CRP and ESR in PJI diagnosis. Moreover, CPR and CFR exhibited diagnostic performance lower than CRP but similar to ESR in PJI diagnosis. These findings suggest that fibrinogen, CPR, and CFR could serve as supplementary markers for aiding in the diagnosis of PJI.

**Table 2:** Comparison of inflammation, coagulation, ratio of inflammation, and coagulation markers between patients from the two groups

Parameters	PJI group	Aseptic loosening group	*p*-value
CRP	36.76 ± 41.89 mg/L	4.85 ± 8.65 mg/L	<0.001^*^
ESR	57.62 ± 30.97 mm/h	22.08 ± 19.84 mm/h	<0.001^*^
PC/MPV ratio	31.95 ± 15.59	24.41 ± 9.57	<0.001^*^
Fibrinogen	4.90 ± 1.66 μg/L	3.21 ± 0.86 μg/L	<0.001^*^
D-dimer	2.28 ± 2.53 μg/L	1.26 ± 1.30 μg/L	<0.001^*^
CPR	Sum of rank 19,477	Sum of rank 6,174	<0.001^#^
	Mean rank 46.44	Mean rank 66.39	
CDR	28.19 ± 47.07	6.35 ± 20.61	<0.001^*^
CFR	6.77 ± 6.66	1.30 ± 2.13	<0.001^*^
EPR	Sum of rank 18,710	Sum of rank 6,941	<0.001^*^
	Mean rank 140.68	Mean rank 74.63	
EDR	45.32 ± 39.20	25.57 ± 24.83	<0.001^*^
EFR	11.45 ± 5.11	6.24 ± 4.50	<0.001^*^

*Independent-samples *t*-test.

^#^Mann–Whitney *U* test.

**Table 3:** The diagnostic performance of different markers in PJI diagnosis

	AUC	95% confidence interval	Associated criterion	Youden index *J*	Sensitivity (%)	Specificity (%)	Significance level *p* (area = 0.5)
CRP	0.876	0.826–0.916	>5.73	0.6238	84.96	77.42	<0.0001
ESR	0.844	0.790–0.889	>32.00	0.5637	78.95	74.42	<0.0001
PC/MPV ratio	0.652	0.586–0.714	>26.77	0.2736	56.39	70.97	<0.0001
Fibrinogen	0.857	0.805–0.900	>4.15	0.5906	75.19	83.87	<0.0001
D-dimer	0.682	0.617–0.743	>1.26	0.3327	60.15	73.12	<0.0001
CPR	0.854	0.801–0.898	>0.29	0.5874	75.94	82.80	<0.0001
CDR	0.827	0.771–0.874	>4.00	0.5819	79.70	78.49	<0.0001
CFR	0.858	0.806–0.901	>1.70	0.6185	81.20	80.65	<0.0001
EPR	0.792	0.733–0.843	>0.97	0.4422	79.70	64.52	<0.0001
EDR	0.688	0.624–0.748	>25.71	0.3295	60.90	72.04	<0.0001
EFR	0.788	0.729–0.839	>5.90	0.4829	90.23	58.06	<0.0001

**Table 4:** Comparison of ROC curves among different markers in PJI diagnosis

	ESR	PC/MPV ratio	Fibrinogen	D-dimer	CPR	CDR	CFR	EPR	EDR	EFR
CRP	*P* = 0.2106	*P* < 0.0001	*P* = 0.3829	*P* < 0.0001	*P* = 0.0075	*P* = 0.0011	*P* = 0.0014	*P* = 0.0054	*P* < 0.0001	*P* = 0.0059
ESR	—	*P* < 0.0001	*P* = 0.4987	*P* < 0.0001	*P* = 0.7280	*P* = 0.5510	*P* = 0.6272	*P* = 0.0032	*P* < 0.0001	*P* < 0.0001
PC/MPV ratio	*P* = 0.0014	—	*P* < 0.0001	*P* = 0.5512	*P* < 0.0001	*P* < 0.0001	*P* < 0.0001	*P* = 0.4437	*P* = 0.0010	*P* = 0.0054
Fibrinogen	—	—	—	*P* < 0.0001	*P* = 0.9056	*P* = 0.2255	*P* = 0.9724	*P* = 0.0173	*P* < 0.0001	*P* = 0.0180
D-dimer	—	—	—	—	*P* < 0.0001	*P* = 0.0037	*P* < 0.0001	*P* = 0.0076	*P* = 0.9244	*P* = 0.0096
CPR	—	—	—	—	—	*P* = 0.1753	*P* = 0.8869	*P* = 0.1396	*P* = 0.0002	*P* = 0.1379
CDR	*P* = 0.5587	—	—	—	—	—	*P* = 0.0569	*P* = 0.3143	*P* < 0.0001	*P* = 0.2842
CFR	*P* = 0.7815	—	—	—	—	—	—	*P* = 0.0434	*P* < 0.0001	*P* = 0.0407
EPR	—	—	—	—	—	—	—	—	*P* = 0.0016	*P* = 0.8160
EDR	—	—	—	—	—	—	—	—	—	*P* = 0.0025
EFR	—	—	—	—	—	—	—	—	—	—


### 3.3 Performance of combine CRP or ESR with fibrinogen, CPR, or CFR in PJI diagnosis

**Figure 3:**
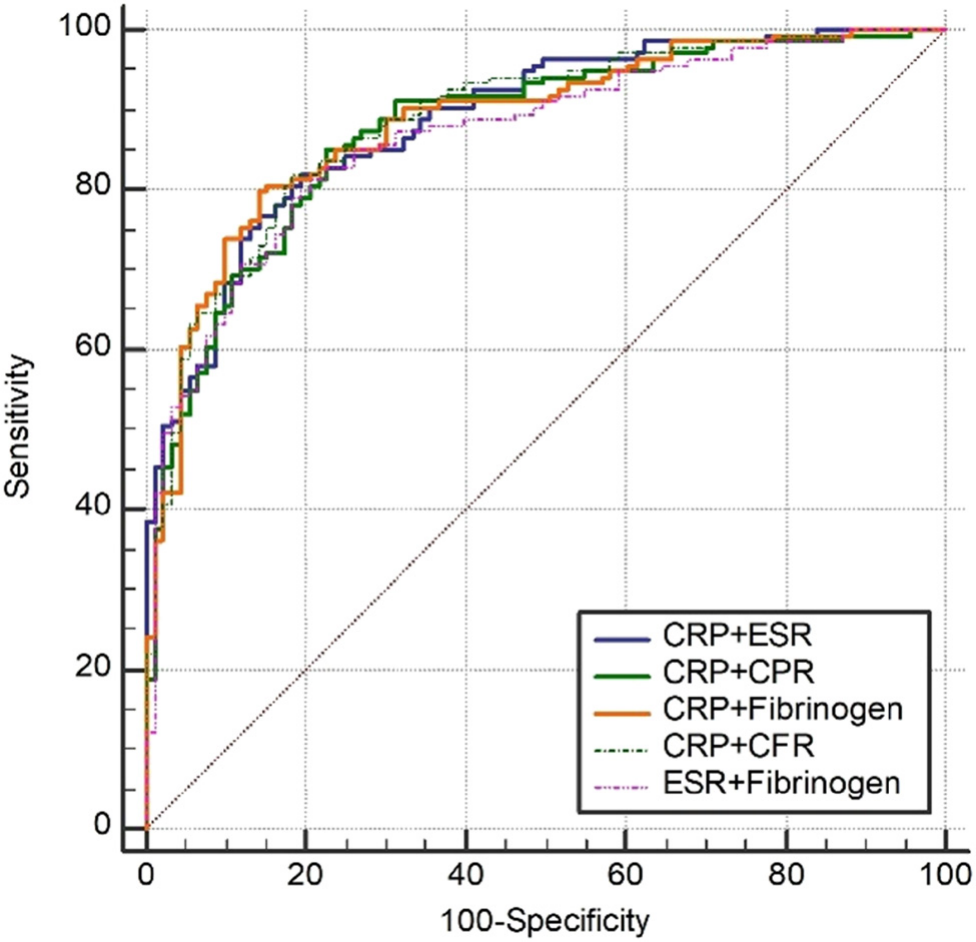
ROC curve of combined CRP or ESR with fibrinogen, CPR, or CFR in PJI diagnosis.

While CRP and ESR are commonly recommended for PJI diagnosis based on the MSIS criteria [21] and the guidelines from the Infectious Diseases Society of America [22], our study suggests that fibrinogen, CPR, and CFR could also be valuable markers for diagnosing PJI. However, not all patients may have complete data available for all these inflammation and coagulation markers at the time of suspected PJI presentation. Therefore, we propose the use of combined CRP or ESR with fibrinogen, CPR, or CFR for PJI diagnosis in cases where all coagulation markers or both CRP and ESR results are not available. Utilizing the ROC curve and AUC, we determined the optimal threshold values, specificity, and sensitivity of these combined markers in PJI diagnosis. As demonstrated in Tables 5 and 6 and Figure 3, the diagnostic performance of combining CRP with CPR, fibrinogen, or CFR, as well as combining ESR with fibrinogen, was comparable to that of combining CRP with ESR. These findings support the use of combined CRP and CPR, combined CRP and fibrinogen, combined CRP and CFR, and combined ESR and fibrinogen for diagnosing PJI.

**Table 5:** Performance of combined CRP or ESR with fibrinogen, CPR or CFR in PJI diagnosis

	AUC	95% confidence interval	Youden index *J*	Sensitivity (%)	Specificity (%)	Significance level *P* (Area = 0.5)
CRP + ESR	0.887	0.838–0.925	0.6271	76.69	86.02	<0.0001
CRP + CPR	0.877	0.826–0.916	0.6238	84.96	74.42	<0.0001
CRP + fibrinogen	0.884	0.835–0.923	0.6572	79.70	86.02	<0.0001
CRP + CFR	0.886	0.838–0.925	0.6368	81.95	81.72	<0.0001
ESR+ fibrinogen	0.867	0.815–0.908	0.6077	81.20	79.57	<0.0001

**Table 6:** Comparison of ROC curves among different markers in PJI diagnosis

	CRP + CPR	CRP + fibrinogen	CRP + CFR	ESR + FIB
CRP + ESR	*P* = 0.3819	*P* = 0.8175	*P* = 0.9797	*P* = 0.1206
CRP + CPR	—	*P* = 0.5402	*P* = 0.1313	*P* = 0.6338
CRP + fibrinogen	—	—	*P* = 0.8199	*P* = 0.1448
CRP + CFR	—	—	—	*P* = 0.2542
ESR + fibrinogen	—	—	—	—

## 4 Discussion

While traditional inflammation markers such as CRP and ESR are commonly utilized for PJI diagnosis, and individual blood coagulation markers like D-dimer, PC/MPV ratio, and fibrinogen have shown promise in identifying PJI, accurately diagnosing PJI based solely on limited inflammation or coagulation markers during the initial consultation remains challenging. Enhancing PJI diagnostic precision with restricted inflammation or coagulation data necessitates further investigation. In our study, we not only established the utility of fibrinogen, CPR, and CFR as standalone markers for PJI diagnosis but also proposed that combining CRP with fibrinogen, CFR, or ESR could serve as novel auxiliary markers for enhancing PJI diagnosis accuracy.

Since Shahi et al. first highlighted the potential of D-dimer as a blood coagulation marker for diagnosing PJI in 2017 [7], there has been a surge of studies investigating the role of coagulation markers in PJI diagnosis over the past five years. While the significance of fibrinogen and FDP in PJI diagnosis has been widely acknowledged across various studies [8,13,15,16],the utility of D-dimer and PC/MPV ratio in PJI diagnosis remains a topic of debate [14,17,18,23–26]. In our study, we conducted a comparative analysis of D-dimer, PC/MPV ratio, and fibrinogen in PJI diagnosis against CRP and ESR. Our findings revealed that the AUC of fibrinogen in PJI diagnosis was comparable to that of CRP and ESR, whereas the AUC of D-dimer and PC/MPV ratio in PJI diagnosis was lower than that of CRP and ESR. These results suggest that fibrinogen could serve as a valuable new auxiliary marker for PJI diagnosis, while D-dimer and PC/MPV ratio may not be as effective in this regard.

Diagnosing PJI accurately can be challenging when patients with suspected PJI exhibit limited inflammation or coagulation markers. While individual inflammation or coagulation markers can aid in PJI diagnosis, the potential of the inflammation-to-coagulation marker ratio or combining CRP or ESR with fibrinogen, CPR, CDR, or CFR for PJI diagnosis remains underexplored [20]. In this study, we discovered that the performance of CPR and CFR in PJI diagnosis was similar to that of ESR, and CPR and CFR can be used as new auxiliary markers for PJI diagnosis. Then, we found that AUC of combined CRP and CPR, combined CRP and FIB, combined CRP and CDR, combined CRP and CFR, combined ESR and fibrinogen in PJI diagnosis were similar with combined CRP and ESR in PJI diagnosis, combined CRP and CPR, combined CRP and FIB, combined CRP and CDR, combined CRP and CFR, combined ESR and fibrinogen can be used as for PJI diagnosis.

## 5 Study limitations

However, our study has several limitations: (1) being retrospective, it may be subject to inherent recall and reporting biases; (2) being single-center, a multicenter study would be beneficial to validate our findings; (3) exclusion criteria such as visible ecchymosis, recent dislocation or trauma, hematoma, hypercoagulation disorder, prosthetic heart valve, systemic inflammatory disease, and tumor may have limited the generalizability of our conclusions; (4) the sample size of 226 patients in our study is relatively small, warranting further investigation with a larger cohort; and (5) this study proposes a methodology based on statistical analysis to further evaluate clinical data with the aim of providing new insights into the diagnosis of PJI. However, we recognize that the application of these methods requires some statistical background and skills, which may pose a challenge to some clinicians. While our methods are theoretically innovative and potentially clinically applicable, they may encounter operational complexity in practical application. Therefore, exploring collaboration with experts in the fields of computer science and biostatistics in future studies to develop an algorithm and corresponding application that can accurately predict the probability of a PJI diagnosis would greatly improve diagnostic accuracy and provide clinicians with stronger decision support.

## 6 Conclusions

**Figure 4:**
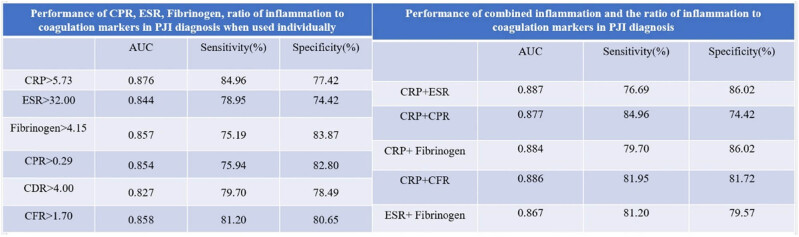
Graphical representation of different markers in PJI diagnosis.

In this study, our findings suggest the following: 1, fibrinogen, CPR, and CFR can be utilized individually for PJI diagnosis; 2, combined CRP and CPR, combined CRP and fibrinogen, combined CRP and CFR, as well as combined ESR and fibrinogen, demonstrate potential as novel auxiliary indicators for PJI diagnosis (Figure 4). Fibrinogen, CPR, CFR, combined CRP and CPR, combined CRP and fibrinogen, combined CRP and CDR (CRP/D-dimer), combined CRP and CFR, and combined ESR and fibrinogen are potential auxiliary markers that can be considered for PJI diagnosis.

## Abbreviations

PJI: periprosthetic joint infection
CRP: C-reactive protein
ESR: erythrocyte sedimentation rate
PC/MPV: platelet count and mean platelet volume ratio
FDP: fibrin degradation products

## References

[1] Lichtman DM, Bindra RR, Boyer MI, Putnam MD, Ring D, Slutsky DJ, et al. American Academy of Orthopaedic Surgeons clinical practice guideline on: The treatment of distal radius fractures. J Bone Jt Surg Am. 2011;93(8):775–8. 10.2106/JBJS.938ebo 21508285

[2] Parvizi J, Gehrke T. International Consensus Group on Periprosthetic Joint I. Definition of periprosthetic joint infection. J Arthroplasty. 2014;29(7):1331. 10.1016/j.arth.2014.03.009 24768547

[3] Parvizi J, Tan TL, Goswami K, Higuera C, Della Valle C, Chen AF, et al. The 2018 definition of periprosthetic hip and knee infection: An evidence-based and validated criteria. J Arthroplasty. 2018;33(5):1309–14 e2. 10.1016/j.arth.2018.02.078 29551303

[4] Tande AJ, Patel R. Prosthetic joint infection. Clin Microbiol Rev. 2014;27(2):302–45. 10.1128/CMR.00111-13 PMC3993098 24696437

[5] McNally M, Sousa R, Wouthuyzen-Bakker M, Chen AF, Soriano A, Vogely HC, et al. Infographic: The EBJIS definition of periprosthetic joint infection. Bone Jt J. 2021;103-B(1):16–7. 10.1302/0301-620X.103B1.BJJ-2020-2417 PMC7954145 33380197

[6] Perez-Prieto D, Portillo ME, Puig-Verdie L, Alier A, Martinez S, Sorli L, et al. C-reactive protein may misdiagnose prosthetic joint infections, particularly chronic and low-grade infections. Int Orthop. 2017;41(7):1315–9. 10.1007/s00264-017-3430-5 28321490

[7] Shahi A, Kheir MM, Tarabichi M, Hosseinzadeh HRS, Tan TL, Parvizi J. Serum D-dimer test is promising for the diagnosis of periprosthetic joint infection and timing of reimplantation. J Bone Jt Surg Am. 2017;99(17):1419–27. 10.2106/JBJS.16.01395 28872523

[8] Huang JC, Chen X, Qiang S, Zheng WD, Zheng J, Jin Y. Exciting performance of plasma fibrinogen in periprosthetic joint infection diagnosis. Orthop Surg. 2021;13(3):812–6. 10.1111/os.12964 PMC8126952 33719200

[9] Shi W, Wang Y, Zhao X, Yu T, Li T. CRP/albumin has a promising prospect as a new biomarker for the diagnosis of periprosthetic joint infection. Infect Drug Resist. 2021;14:5145–51. 10.2147/IDR.S342652 PMC8664647 34908848

[10] Christopher ZK, McQuivey KS, Deckey DG, Haglin J, Spangehl MJ, Bingham JS. Acute or chronic periprosthetic joint infection? Using the ESR CRP ratio to aid in determining the acuity of periprosthetic joint infections. J Bone Jt Infect. 2021;6(6):229–34. 10.5194/jbji-6-229-2021 PMC8209584 34159047

[11] Yigit S, Akar MS, Sahin MA, Arslan H. Periprosthetic infection risks and predictive value of C-reactive protein/albumin ratio for total joint arthroplasty. Acta Biomed. 2021;92(4):e2021324. 10.23750/abm.v92i4.10995 PMC8477118 34487091

[12] Foley JH, Conway EM. Cross talk pathways between coagulation and inflammation. Circ Res. 2016;118(9):1392–408. 10.1161/CIRCRESAHA.116.306853 27126649

[13] Xu H, Xie J, Huang Q, Lei Y, Zhang S, Pei F. Plasma fibrin degradation product and D-dimer are of limited value for diagnosing periprosthetic joint infection. J Arthroplasty. 2019;34(10):2454–60. 10.1016/j.arth.2019.05.009 31155460

[14] Paziuk T, Rondon AJ, Goswami K, Tan TL, Parvizi J. A novel adjunct indicator of periprosthetic joint infection: Platelet count and mean platelet volume. J Arthroplasty. 2020;35(3):836–9. 10.1016/j.arth.2019.10.012 31759801

[15] Wu H, Meng Z, Pan L, Liu H, Yang X, Yongping C. Plasma fibrinogen performs better than plasma D-dimer and fibrin degradation product in the diagnosis of periprosthetic joint infection and determination of reimplantation timing. J Arthroplasty. 2020;35(8):2230–6. 10.1016/j.arth.2020.03.055 32376167

[16] Bin G, Xinxin Y, Fan L, Shenghong W, Yayi X. Serum fibrinogen test performs well for the diagnosis of periprosthetic joint infection. J Arthroplasty. 2020;35(9):2607–12. 10.1016/j.arth.2020.04.081 32446625

[17] Huang J, Zhang Y, Wang Z, Dong Y, Zhao Y, Zheng J, et al. The serum level of D-Dimer is not suitable for distinguishing between prosthetic joint infection and aseptic loosening. J Orthop Surg Res. 2019;14(1):407. 10.1186/s13018-019-1461-x PMC6884846 31783874

[18] Li C, Margaryan D, Ojeda-Thies C, Perka C, Trampuz A. Meta-analysis of serum and/or plasma D-dimer in the diagnosis of periprosthetic joint infection. J Orthop Surg Res. 2020;15(1):298. 10.1186/s13018-020-01808-1 PMC7409706 32762703

[19] Sahin E, Karaismailoglu B, Ozsahin MK, Guven MF, Kaynak G. Low value of platelet count to mean platelet volume ratio to diagnose chronic PJI: A case control study. Orthop Traumatol Surg Res. 2021;107(4):102899. 10.1016/j.otsr.2021.102899 33774191

[20] Wu Y, Sun K, Liu R, Wu L, Zeng Y, Li M, et al. C-reactive protein/albumin and C-reactive protein/fibrinogen ratios for the diagnosis of periprosthetic joint infection in revision total joint arthroplasty. Int Immunopharmacol. 2023;115:109682. 10.1016/j.intimp.2023.109682 36623413

[21] Parvizi J, Zmistowski B, Berbari EF, Bauer TW, Springer BD, Della Valle CJ, et al. New definition for periprosthetic joint infection: From the Workgroup of the Musculoskeletal Infection Society. Clin Orthop Relat Res. 2011;469(11):2992–4. 10.1007/s11999-011-2102-9 PMC3183178 21938532

[22] Osmon DR, Berbari EF, Berendt AR, Lew D, Zimmerli W, Steckelberg JM, et al. Diagnosis and management of prosthetic joint infection: clinical practice guidelines by the Infectious Diseases Society of America. Clin Infect Dis. 2013;56(1):e1–25. 10.1093/cid/cis803 23223583

[23] Zmistowski B, Chang M, Shahi A, Nicholson T, Abboud J, Lazarus M, et al. Is D-dimer a reliable serum marker for shoulder periprosthetic joint infection? Clin Orthop Relat Res. 2021;479(7):1447–54. 10.1097/CORR.0000000000001774 PMC8208387 33929986

[24] Yan J, Xie K, Jiang X, Han X, Wang L, Yan M. D-dimer for diagnosis of periprosthetic joint infection: A meta-analysis. J Orthop Sci. 2021;26(6):1036–42. 10.1016/j.jos.2020.09.015 33127211

[25] Lu G, Li T, Ye H, Liu S, Zhang P, Wang W. D-dimer in the diagnosis of periprosthetic joint infection: A systematic review and meta-analysis. J Orthop Surg Res. 2020;15(1):265. 10.1186/s13018-020-01761-z PMC7364596 32677991

[26] Xu H, Xie J, Yang J, Chen G, Huang Q, Pei F. Plasma fibrinogen and platelet count are referable tools for diagnosing periprosthetic joint infection: A single-center retrospective cohort study. J Arthroplasty. 2020;35(5):1361–7. 10.1016/j.arth.2019.12.015 31899088

